# Cultural sensitivity in brain death determination: a necessity in end-of-life decisions in Japan

**DOI:** 10.1186/s12910-021-00626-2

**Published:** 2021-05-13

**Authors:** Yuri Terunuma, Bryan J. Mathis

**Affiliations:** 1 School of Medicine, University of Tsukuba Medical School, 1-1-1 Tennodai, Tsukuba, Ibaraki Japan; 2 International Medical Center, University of Tsukuba Affilated Hospital, 2-1-1 Amakubo, Tsukuba, Ibaraki 305-8575 Japan

**Keywords:** Brain injury, End-of-life issues, Health policy, Law, Culture

## Abstract

**Background:**

In an increasingly globalized world, legal protocols related to health care that are both effective and culturally sensitive are paramount in providing excellent quality of care as well as protection for physicians tasked with decision making. Here, we analyze the current medicolegal status of brain death diagnosis with regard to end-of-life care in Japan, China, and South Korea from the perspectives of front-line health care workers.

**Main body:**

Japan has legally wrestled with the concept of brain death for decades. An inability to declare brain death without consent from family coupled with cultural expectations of family involvement in medical care is mirrored in other Confucian-based cultures (China and South Korea) and may complicate care for patients from these countries when traveling or working overseas. Within Japan, China, and South Korea, medicolegal shortcomings in the diagnosis of brain death (and organ donation) act as a great source of stress for physicians and expose them to potential public and legal scorn. Here, we detail the medicolegal status of brain death diagnosis within Japan and compare it to China and South Korea to find common ground and elucidate the impact of legal ambiguity on health care workers.

**Conclusion:**

The Confucian cultural foundation of multiple Asian countries raises common issues of family involvement with diagnosis and cultural considerations that must be met. Leveraging public education systems may increase awareness of brain death issues and lead to evolving laws that clarify such end-of-life issues while protecting physicians from sociocultural backlash.

## Background

Modern, Western-style medicine requires that the three core principles of informed consent regarding patient care (disclosure of information, competency of patient to decide, and voluntary decisions) be present in every aspect of care [1]. This dictates that physicians must provide adequate information, without coercion, on the risks, benefits and alternatives to procedures and interventions (medical or surgical) but the patient must also completely understand this information and make decisions based on free will. Japanese regulatory agencies, like those in Western countries, have adopted the legal codification of these principles within the International Conference on Harmonisation’s *Guidelines for Good Clinical Practice* framework [2]. Even so, Japanese physicians must still give precedence to family consent over individual autonomy. Although family consent follows similar guidelines to individual consent, it places greater weight on choices made by families versus individuals [3]. This disconnect between the adoption of international principles and actual clinical practice within Japan stems from the Confucian basis of the Japanese culture, namely the cultural norm that authorities (physicians, families) know better than the individual but also that the family is responsible to support the patient throughout treatment [4, 5]. This gives great weight to the family members in deciding the fate of a family member with regard to brain death diagnosis. Why would this be so different than the self-centered Western thinking?

In Asia, as in the rest of the world, legal and informal control of medical activities are driven by cultural perceptions regarding the role of the patient, doctor, and family (or society) in deciding on both provision and removal of care. Taking a holistic, theoretical view of Asian culture and its Confucian roots, we find that, by the standards of Gelfand and Uz, Japan, China, and South Korea are themselves tight and intolerant societies, in which culture and behavior is rigidly defined while deviations by individuals from expected norms are punished on a societal level [6, 7]. This is representative of the Asian paradigm, where cultural analyses conducted from an *etic* viewpoint find that, paradoxically, wealthy Asian countries with urbanized development and globalized business strategies tend to be both tight and intolerant with their own citizens with regard to maintaining norms, reflective of the poorer, agriculturally based societies they evolved from [8]. Additionally, due to the standardization of Chinese writing and its adoption since ancient times by modern, industrially advanced countries (such as South Korea and Japan), homographic concepts explained in *kanzi* (Chinese letters), such as “death” or “duty,” are commonly understood across linguistic barriers since the intent or meaning of the characters does not change [8, 9]. Therefore, because of this common heritage, Asian countries cannot collectively escape the phenomenon whereby rapid industrialization/globalization outstrips their ability to evolve ossified traditions, mores, and norms to keep pace. Within an *emic* view of each society, however, diverse interactions within the social constructs generated by a rigid positional orthodoxy could create deviations from a common ancestry as seen in Japanese, South Korean and Chinese medical fields where traditional Chinese medicine (adopted from ancient times within all 3 countries) is used at different ratios as an adjunct therapy for Western-style medicine [8]. This indicates that, while cultural and moral norms derived from Confucianism may more slowly change, the assimilation of Western concepts on a technical level happens at a unique rate for each Asian society.

This concordant opposition of social and medical forces introduces conflict at the level of individual physicians within Asian countries since medical training is based on Western-style medicine. Integration of Western medicine thus forms the core competency of all licensed physicians in Asia to drive medical advancements and standards of care. But, since Western-style medical training emphasizes the autonomy of the individual, Asian physicians must somehow reconcile this requirement with their inherent cultural training to respect the family above all else [10, 11]. In this lies the conundrum of end-of-life care in Asia: can a doctor do what may be medically unsound to fulfill cultural/societal expectations or must a doctor do what Western medicine says is right and endure the resultant sociocultural backlash? The ability to decide these issues at the individual level is what Park and colleagues [12] referred to as the classic “self-construal” concept of Markus and Kitayama when they attempted to measure the independent/interdependent status of Koreans and Japanese to catalogue cultural differences at the societal level with regard to subjective well being [12]. They found that Japanese tend to be “self-focused relational selves,” primarily occupied with how others perceive them, and this was also found in older South Koreans [12]. This type of cultural integration would increase pressure on Japanese and older South Korean doctors to conduct themselves in a manner meeting with social approval, up to and including end-of-life decisions. Younger South Koreans, on the other hand, were more “other-focused relational selves,” forming bonds with others and being attuned to them; a possibility stemming from their temporal distance from the disruptive Korean War [12]. However, even young South Korean doctors would be expected to conform to family-centered thinking due to parental influence and, as the average age of South Korean workers is 42, the younger generation will be dealing with the more rigid, traditional culture for the foreseeable future [13]. Likewise, a person is viewed as a “relational self” in China, and physicians are customarily driven to work for the good of the social relationships that support such personas [14, 15]. It can thus be said that doctors from modern Asian societies struggle with conflicts between societal expectations (the power of the family) and the indications of Western medicine (founded by an individualistic culture alien to Confucian thought).

Clinical protocols and laws regarding brain death are thus affected in Japan and other Asian countries by this complex interplay between cultural and social expectations. Despite implementing proper laws outlining brain death, the Japanese health system still struggles with the diagnostic aspect. Underlying this issue is a complex mix of legal, financial, and cultural aspects of Japanese society. Here, we review the current situation regarding brain death determination with Japan as a primary example and include comparisons to other Confucian-based Asian countries.

## Japanese perspective on brain death

### Legal status

The Organ Transplant Law was introduced in 1997 as the first official Japanese law equating brain and legal death. Although legally revised twice, diagnosed brain death still exists solely in the context of organ donation. Furthermore, because familial consent is still legally required for brain death declaration, the actual clinical context has remained unchanged and Japanese laws still permit familial overriding of the patient’s wishes. Thus, even if brain death is suspected, physicians must consult patient families before any testing is initiated since cultural Confucianism remains the foundation for law-making bodies in Japan.

These laws reflect the *etic* perspective that Japanese society is intolerant of any deviations from cultural and societal norms. When Dr. Juro Wada was accused of murder and fraud in Japan’s first heart transplantation from a brain-dead donor in 1968, he was heavily criticized and even arrested for murder due to the novelty of the concept of brain death at the time [16]. Although later acquitted, claims of corrupt handling of both the donor and the brain death determination process were extensively publicized. Apprehension towards brain death and its appropriate application in medical practice thus persists among the general public to this day. This historical controversy, combined with the self-focused relational perspectives that prevail among Japanese physicians, thus compel acquiescence to the cultural expectation of familial wishes. The Organ Transplant Law therefore serves as a compromise by validating brain death, but only in the context of organ donation and with the family’s permission.

### Working practice

The Japanese Ministry of Health, Labour and Welfare established national guidelines for brain death determination in 1997 [17, 18]. These guidelines stipulate specific criteria for brain death diagnosis and are among the strictest in the world. The Japanese guidelines notably require confirmation of electrocerebral inactivity on electroencephalogram [18]. Electroencephalography, though readily available, requires technical knowledge and special facilities to maintain accuracy. Hence, a shortage of capable staff members and facilities have ironically hindered its integration into routine medical practice in Japan.

The Japanese health care system and its affordability could significantly hamper brain death diagnosis since universal health coverage is both affordable and scaled to illnesses, age, and socioeconomic status. Overall lower medical costs create a family expectation to sustain a comatose patient until the family reaches a decision.

Japanese cultural aversion to organ donation also delays brain death diagnosis. In addition to the 1968 Wada incident, brain death education in Japan is inadequate [19]. Moreover, Japanese culture, deeply rooted in Confucianism, expects the family to care for its ill and even death is a social event involving family. Thus, physicians are culturally expected to prioritize family wishes over patient wishes [20]. Furthermore, brain death in an otherwise healthy body conflicts with the holistic interpretation of the body and soul that is prevalent in Japan, invoking reluctance towards acknowledgement of brain death as true death [21]. As a result, many Japanese prefer to continue end-of-life support until cardiovascular death.

### Effect on health care workers

According to the Japanese legal brain death criteria, only physicians in certain specialties can diagnose brain death [18]. Additionally, only designated donor hospitals may diagnose brain death [18]. Thus, this responsibility is dispersed unequally among medical specialties and facilities in Japan. Even so, donor hospitals lack various support systems, including socioeconomic and psychological support, for medical staff [22].

Health care workers are legally and culturally obliged to present brain death diagnosis and subsequent organ donation as an option to the family before initiating diagnostic testing. However, as brain death is legally considered as solely in the context of organ donation in Japan, the physician cannot proceed with a brain death diagnosis if the patient’s family refuses organ donation [23]. This creates a situation where potentially brain-dead patients can be kept on life support indefinitely until the heart stops.

The issue of sustaining the body of a brain-dead patient until cardiovascular death continues to divide Japanese doctors. A general lack of end-of-life discussion due to the prevailing cultural attitude of cardiovascular death as definitive among the Japanese is a major propagator of this dichotomy. In a 2017 survey conducted by the Japanese Ministry of Health, Labour and Welfare [24], only 39.5% of the general population had discussed end-of-life preferences with others despite the establishment of guidelines for advance care planning. More than 94% of these discussions were with family members, whereas 4.2% were with medical staff. Likewise, a mere 8.1% of the general population had completed advance directives despite the 66.0% support rate. Minimal recognition of advance care planning among the general public and the lack of legislation backing advance directives have been attributed to these low rates of action. Without adequate end-of-life discussion, care decisions are ultimately made by physicians and families instead of the patients themselves. Additionally, as Japan lacks guidelines regarding life-sustaining treatment and systems that legally protect physicians when withholding or withdrawing care from patients, cases exist where physicians were publicly criticized for discontinuing life support and violating sociocultural norms [25]. As a result, Japanese physicians are pressured into continuing life-sustaining treatment out of fear of litigation or public scorn regardless of medical considerations. To counter this, hospitals have begun multidisciplinary conferences to discuss these medical issues but nationwide consensus on care for the potentially brain-dead has yet to be reached.

## Comparisons to other Asian countries

### China

#### Legal status

In 2013, the Brain Injury Evaluation Quality Control Center published criteria and practical guidelines for brain death diagnoses. Physicians are also required to receive training and certification by the Brain Injury Evaluation Quality Control Center to diagnose brain death [26, 27]. However, China lacks concrete laws pertaining to brain death and its diagnosis [28].

This delay in introduction of concrete legislation on brain death can be attributed to the *etic* viewpoint within Chinese society. Despite multiple attempts by various organizations in China to legalize brain death, all efforts were rejected by the Chinese Ministry of Health [29]. This history upholds the paradigm that Asian societies, including China, are reluctant to incorporate ideas that do not follow traditional ideals and notions. From an *emic* standpoint, low levels of health literacy contribute to how relatively little-known brain death is among the general public in China [29], leading to a paucity of popular support for brain death regulations.

#### Working practice

Family refusal is a major non-medical factor that can preclude the diagnostic process [27]. In China, family members can refuse brain death testing on a patient suspected to be brain dead since it is not yet recognized as a legal form of death. Due to the “relational self” perspective and resultant emphasis on social relationships that prevails in China, physicians are obliged to respect the desires of the family when treating the patient.

Chinese traditions also delay the incorporation of brain death into medical practice. The Chinese cherish filial piety and are culturally driven to look after ill family members for as long as possible. Traditional Chinese culture also portrays death as a life event involving family [28]. According to Taoism, the native belief of China, the mind and body are closely linked [28]. China, like Japan, culturally characterizes death by the cessation of breathing and cardiovascular activity and incorporates family wishes into end-of-life decisions [30]. The cultural importance of familial decisions and lack of laws legitimizing brain death serve as obstacles to brain death diagnosis in China.

### South Korea

#### Legal status

Laws validating brain death and protocols for brain death determination have been established by the national government [31]. However, brain death exists solely as a premise for organ donation in South Korea. Family consent, though non-essential for brain death testing, is needed for organ donation. Therefore, patients suspected to be brain dead ultimately cannot be diagnosed as such unless their family members consent to the entire organ donation process.

Like Japan, brain death is paired with organ donation in South Korea. The mandatory family consent included in the law reflects the *etic* aspect of South Korean society to respect the cultural norms of honoring family wishes when making important medical decisions. Since the self-focused relational self is still predominant among both working-age and elderly Koreans, this view upholds the principles that constitute laws regarding brain death diagnosis.

#### Working practice

According to the South Korean protocol, brain death can only be declared after a brain death determination committee, which includes both medical and non-medical members, reviews the brain death examination results and reaches a unanimous decision [32]. Thus, it is ultimately up to a committee rather than individual physicians to determine whether or not a patient can be declared brain dead.

Death is a topic that is rarely discussed and often avoided in Korean traditions, delaying the acceptance of brain death into medical practice [33, 34]. Additionally, Koreans generally eschew life support if terminally ill in accordance with the Confucian ethos regarding death as a natural process [33]. However, Confucianism also values filial piety and family involvement in decision making. This, combined with an overall lack of advanced care planning, can lead to situations where life-sustaining treatment of brain-dead patients is continued until cardiovascular death, even if contrary to the patient’s true and evoked wishes. Therefore, although brain death is legally defined as a form of death in South Korea, traditional Korean beliefs may impede its integration into medical practice and alter medical decision making.

## Conclusions

Japanese beliefs on matters of brain death hinder diagnosis and are heavily impacted by legal, cultural and financial factors. In terms of legislation, Japanese laws mandate patient family consent for the diagnosis of brain death. Strict guidelines on brain death diagnosis also complicate the procedure due to inadequate staff and facilities. In addition, brain death exists as a definition of death only in the context of organ donation. In terms of finance, the Japanese medical system facilitates longer hospital stays. In terms of culture, Japanese culture is family-centered rather than individualistic. Because Japanese culture endorses caring for family, many prefer to delay death until cardiovascular death. Physicians are culturally expected to respect family wishes over patient wishes and legally compelled to do so. Finally, the Japanese see the mind and body as inseparable. Therefore, “brain death,” or cessation of brain function independent of other body functions, is inconsistent with traditional Japanese values. For these reasons, Japanese society does not readily accept brain death [21]. At this time, Japan (and other Asian countries) seem to be where the United States was in the 1970s and 1980s, namely grappling with medical definitions of death that are complicated by religious and cultural standards even in the face of legal definitions (again, centered around organ donation) from a Presidential Commission [35]. However, while Asia takes cues from what it believes is the “settled” Western definition of brain death, Western philosophy and bioethics have been questioning this standard since it was made. A representative example from the philosophical standpoint was the mention by Bayertz (1992) of the “denaturalization” of death due to technological blurring of the physiological line that had previously existed [36]. Additionally, the worth or value of harvestable organs (functionalization) and normalization of definitions (homogenization) create a moral conundrum in which human life becomes a commodity of value (organs) that require a commercialization process (homogenization) based on the intentional setting of a brain death criterion for the commodity to be available (denaturalization) [36]. Thus, the difficulty of diagnosing brain death becomes an issue of morality in the Western sense and family determinism in the East but with the same ambiguous and risky path for the physician(s) in charge of care. In the case of the West, bioethics has been grappling with the issue of mechanical life support during brain death for decades, as seen in 2005 where Veatch argues that loss of some brain functions does not mean the brain itself is “dead” and in 2018, where Singer asserts that medical science cannot distinguish true brain death from a minimal state of consciousness [37, 38]. Here, we come to the crux of the brain death issue in Asia, namely that Western medicine, which itself has not fully decided on brain death, has issued guidelines taught to Asian doctors that conflict with traditional views of life, illness, medical care, death, and families that have been cemented into Confucian traditions. Taken together, this creates the unenviable position for doctors in Asia of having to make medical decisions based on social and cultural expectations but without the legal support needed to confidently do so.

The lack of legal support may be due to the fact that these views, as detailed above, are shared by many patients from other Confucianist countries, including China and South Korea, while other Asian countries (Southeast or Western Asia) may have different perspectives and legal implications concerning brain death. Nonetheless, cultural competence education (East vs. West) for Asian doctors could raise discussion of this issue and increase the overall knowledge, skills, and attitude of health professionals, leading to higher patient and family satisfaction [39, 40]. Integrating such cultural (and medical) education within the public school systems of each Asian country would also increase both awareness of end-of-life directives as well as provide the popular support needed to firmly establish brain death laws that clarify the issue (to the extent that such an issue can be clarified to the satisfaction of that society) and protect physicians from social and cultural backlash.

The future of medical ethics (especially with regard to brain death) within Asian countries is ever evolving but faces challenges to overcome the inertial resistance of older generations inculcated with Confucian beliefs. With the influx of Western entertainment, education and religion, norms and mores may become more individualistic and, over time, perceptions of death and the role of patients, doctors and families may also shift. However, until then, individual doctors in Japan and Asia must continue to walk the tightrope between socio-legal expectations and medical best practice.

## Data Availability

Not applicable.
